# Lessons from history: viral surveillance in 1940s East Africa: Epidemiological notes on some viruses isolated in Uganda, G. W. A. Dick, Transactions of the Royal Society of Tropical Medicine and Hygiene, 1953;47(1):13–48

**DOI:** 10.1093/trstmh/try086

**Published:** 2018-09-18

**Authors:** Lauren B Carrington, Bridget Wills

**Affiliations:** 1 Oxford University Clinical Research Unit, Ho Chi Minh City, Vietnam; 2 University of Oxford, Oxford, UK

**Keywords:** Africa, emerging viruses, Uganda, virus surveillance, West Nile virus, Yellow fever virus

In recent years a series of major outbreaks of emerging/re-emerging viral infections, including severe acute respiratory syndrome in 2003, Middle East respiratory syndrome–coronavirus in 2012, Ebola in 2014 and Zika in 2015, have caused widespread concern and sparked international public health emergencies. Slowly the world has come to realise that coordinated international leadership and a holistic approach are essential to ensure that timely and effective responses are put in place to counter these rapidly evolving threats to human health.

In such circumstances the importance of prior knowledge about the infectious agent cannot be underestimated; having some information about clinical epidemiology and disease pathogenesis in humans and any reservoir hosts and, where relevant, about the vector and associated transmission cycles is invaluable. Here we highlight a remarkable publication in Transactions of the Royal Society of Tropical Medicine and Hygiene that followed a presentation to the society in December 1952 by G. W. A. Dick, in which he summarized a body of research carried out to characterise 10 viruses, 8 of them novel, at the Yellow Fever Research Institute in Uganda during the late 1930s and 1940s (Table 1)^1^. The novel detections were all incidental findings associated with a programme of systematic surveillance and empirical research on yellow fever, which had been instituted in 1936 after yellow fever was declared a regional research priority following an epidemic. For each virus Dick provides detailed evidence regarding infection and host permissiveness in man, monkeys and mosquitoes and describes what researchers at the institute had established concerning epidemiology and transmission dynamics, as well as presenting the methodology used to obtain this evidence. Throughout the article the author considers all evidence conservatively, carefully reflects on the implications and poses interesting questions for future research. Although we now know a great deal more about some of these viruses, including the potential for considerably greater virulence than initially thought, we have failed to extend our knowledge about numerous others. This engaging review puts into perspective how far we have come in the field of tropical medicine, while also highlighting how far we still have to go.

**Table 1.** Summary of viruses isolated in Uganda between 1937 and 1947

			Virus isolation		
Virus name	First detection in Uganda	Year of first isolation	Humans	Sentinel monkeys	Mosquitoes
Yellow fever virus	*		**✓**	**✓**	**✓**
Rift Valley fever virus	*		**✓**		**✓**
West Nile virus	West Nile region	1937	**✓**		
Bwamba virus	Bwamba region	1937	**✓**		**✓**
Semliki Forest virus	Bwamba region	1942			**✓**
Ntaya virus	Bwamba region	1943			**✓**
Bunyamwera virus	Bwamba region	1943			**✓**
Mengo virus	Entebbe region	1946	**✓**	**✓**	**✓**
Zika virus	Entebbe region	1947		**✓**	**✓**
Uganda S virus	Bwamba region	1947			**✓**

*Yellow fever and Rift Valley fever viruses were discovered for the first time in countries other than Uganda.

Even in 1952, Dick emphasised how critical it was to take every opportunity to study the natural history of these novel agents during outbreaks, despite the pressure of maintaining effective public health services. Nowadays we are familiar with the concepts of pandemic preparedness—for example, advance training of outbreak response units, development of efficient quarantine procedures, emergency vector control measures and field vaccination programs and capacity building through community engagement to modify behaviour and reduce transmission risk. Alongside these measures there is increasing recognition that research can, and indeed should, be carried out in such circumstances, albeit with careful attention to the ethical constraints inherent in such work.

Dick expressed a number of other remarkably prescient ideas in this monograph. For example, he observed that several of the viruses appeared to cause symptomatic infection infrequently in the African population and questioned whether this really reflected low circulation. He postulated an alternative theory that people might have developed cross-reactive, neutralizing antibodies through a series of exposures to related viruses—that is, he suggested the presence of immunological relationships between different viruses in human hosts. While we are now very familiar with cross-reactivity of antigens and antibodies produced by related viruses, particularly among the flaviviruses,^2^ there remain many unanswered questions. Indeed, given the suitable climate and susceptible vector populations in the Southeast Asian region, the reason why yellow fever is not endemic in the region remains a mystery.

Dick also questioned whether virus pathogenicity could be altered under different conditions, speculating about the contribution of different vectors to virus transmissibility and whether one vector species might favour multiplication of more virulent members of the virus population over another. Today, several novel mosquito control strategies are being developed and deployed around the world, such as *Wolbachia*-based and genetically modified mosquito strategies.^2,3^ In implementing these programmes, both direct and indirect selective pressures are applied to the mosquitoes and the viruses they carry, and the possibility that these pressures may indeed favour survival of more virulent members of the virus population should not be ignored.

The expanding global population continues to encroach into forested areas, and changing human behaviour and movement patterns increase the exposure of naïve groups to novel viruses. It is noteworthy that the eight previously unknown viruses described by Dick were discovered in only a decade of research (that was primarily focused on yellow fever), highlighting the value of active surveillance programmes in both vector/reservoir populations and human cohort studies. The threat that novel viruses and emerging pathogens present to human health remains as potent now as it was in the first half of the last century, yet science has advanced dramatically both technologically and on a molecular level. What might be achieved today if we applied the same systematic effort? Unfortunately, early warning surveillance systems for known viruses, let alone emerging viruses, remain very limited and most funding mechanisms no longer support sustained, long-term surveillance programmes but rather are focused reactively toward immediate threats. To be properly prepared for the next pandemic, a comprehensive approach will surely be needed—encompassing not only a broad array of immediate outbreak response mechanisms, but also predicated on robust infrastructure development that should include the establishment of long-term viral surveillance systems, particularly in epidemic hotspots.

This article provides a thorough, and yet captivating, overview of research on a number of viruses, both existing and novel, in the 1940s in Uganda. This compilation and interpretation of such a wealth of epidemiological and virological data highlight the magnificent research and surveillance efforts of the Yellow Fever Research Institute. Dick’s advocacy for research on the natural history of emerging viruses during outbreaks and his compelling questions about virus adaptation and evolution still resonate with the reader today. The account provides a welcome reminder of our progress in public health surveillance of emerging infections and what we can do to promote preparedness for future outbreaks.
